# Evaluation of China’s fertility policy based on PMC modeling

**DOI:** 10.3389/fpubh.2025.1533307

**Published:** 2025-02-05

**Authors:** Jie Chen, Yan Gao, Xi Wang

**Affiliations:** ^1^The Experimental School, The Open University of China, Beijing, China; ^2^Jilin Land Planning Research Office, Changchun, China; ^3^School of Management, Changchun Guanghua College, Changchun, China; ^4^Institute of Economic Research, Jilin Academy of Social Sciences, Changchun, China

**Keywords:** fertility policy, text mining, PMC index, policy evaluation, policy optimization

## Abstract

**Background:**

The fertility level has declined to below replacement level in many countries. Hence, evaluating the fertility policies is crucial for policy intervention to achieve sustainable development. However, research on assessing fertility policies remains limited.

**Objective/methodology:**

This study introduces a Policy Maturity and Consistency (PMC) index model based on text mining techniques to analyze 22 fertility policy documents. The analysis model aims to identify policy deficiencies and provide actionable insights for improving future policy frameworks.

**Results:**

Our study shows that, despite a comprehensive design and high consistency, significant gaps remain in areas such as policy objectives and thematic focus. Specific recommendations are proposed to enhance policy effectiveness, including fostering multi-stakeholder collaboration, integrating economic and policy-based support mechanisms, and promoting a shift in reproductive culture.

## 1 Introduction

The global fertility has significantly declined, leading the world toward a low-fertility future (1). Currently, more than half of the world’s countries are below the fertility replacement level (2). Most regions are in transition toward natural population decline, and it is anticipated that by 2,100, only six countries will have above replacement fertility replacement level fertility (3). This demographic shift, characterized by low or negative growth, presents serious challenges to society, the economy, and sustainable development (4). Therefore, it is urgent to address the current fertility situation and promote more balanced population development. Scholars primarily examine fertility from two perspectives: macro and micro. In macro-level research, future fertility rates and birth rates are predominantly predicted using series data (5–7). Conversely, at the micro level, greater emphasis is placed on individuals’ fertility intentions and behaviors (8–10). Nevertheless, the aforementioned perspectives primarily examine future scenarios without considering the influential role of fertility policy. Proactive adjustments to fertility policy serve as an effective strategy to enhance fertility intentions (11, 12). However, some research indicates that population easing policies (QEPP) alone are insufficient to reverse the trend of population decline, highlighting the need for additional focus on public health services (13). Our study employed the PMC quantitative evaluation model, utilizing text mining (TM) techniques, to assess existing fertility policies. This approach holds significant importance for fostering balanced population development. Additionally, it offers a novel research perspective on female fertility.

**Figure 1:**
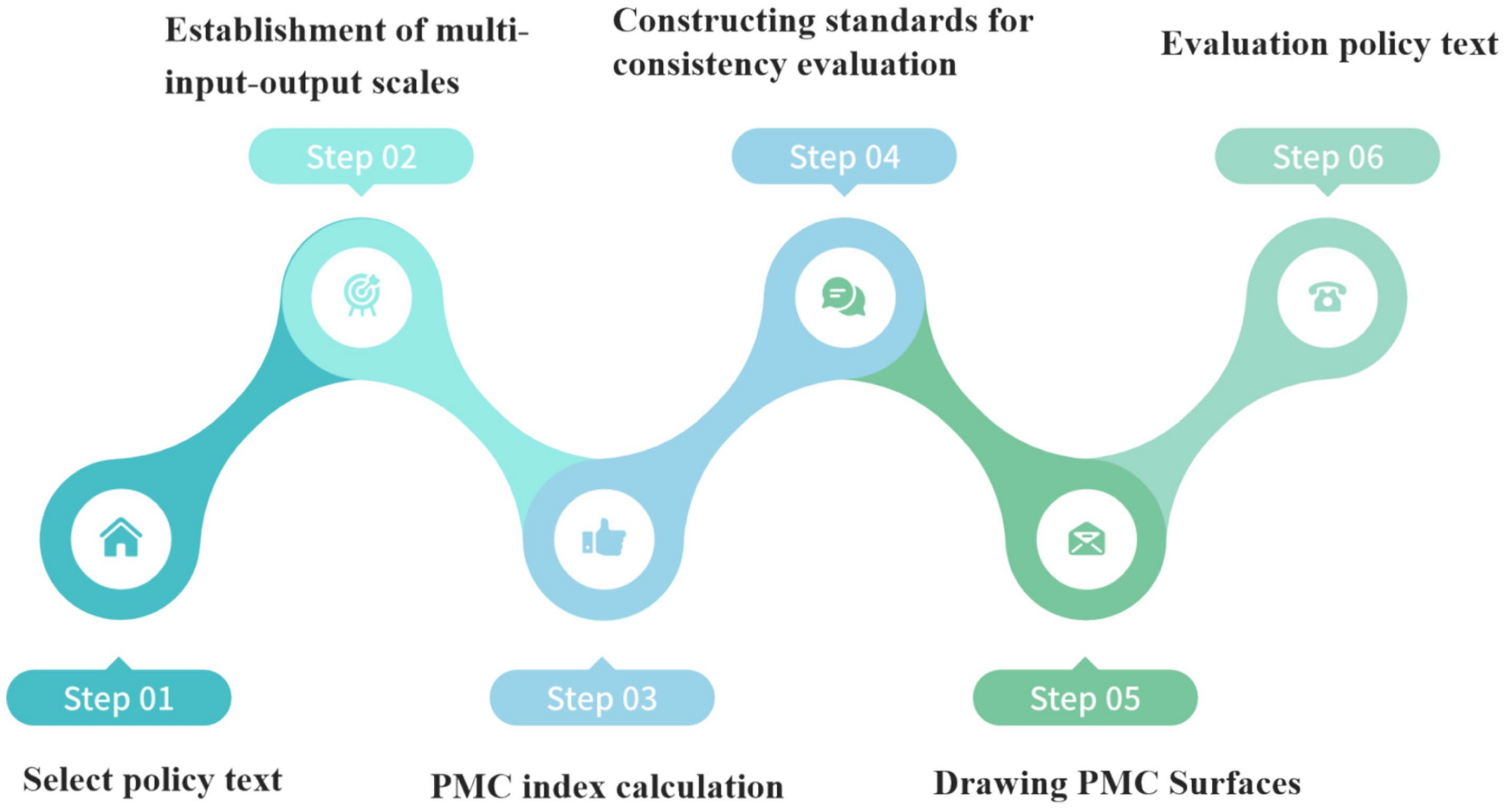
PMC quantitative assessment flowchart.

The PMC quantitative assessment model was proposed by Estrada based on the “Omnia Mobilis” hypothesis (14, 15). The construction of the model involves six key steps: (1) selecting policy texts; (2) constructing multiple input–output tables; (3) calculating PMC indexes; (4) establishing consistency evaluation criteria; (5) plotting PMC surfaces; and (6) evaluating the policy texts against the established criteria, as illustrated in Figure 1. PMC models have gained prominence as evaluation tools in various fields, including energy (16, 17), environment (18, 19), and security (20, 21), particularly in recent years. However, it is important to note that PMC models often suffer from a limited number of evaluations and an excessive reliance on subjective indicators (22).

Based on this analysis, our research identified the shortcomings of fertility policy research and proposed an innovative PMC fertility policy assessment model utilizing text mining techniques. This model constructs a semantic network of policy text keywords through the quantitative assessment of PMC, leveraging the capabilities of text mining. This study provides an in-depth analysis of the relationship between keywords to summarize the core points within the policy text. Additionally, we construct a multi-input–output scale based on these core points to address the excessive subjectivity inherent in the PMC model indicators. Concurrently, we selected 22 policy texts to conduct a comprehensive evaluation of research policies, with the aim of identifying deficiencies in the current fertility policy. This analysis seeks to offer decision-making references for the enhancement of fertility policy texts in the future.

## 2 Research methods

### 2.1 Objects

The study has chosen the Chinese government as the subject for several reasons. First, the Chinese government has experienced a continuous decline in fertility since the introduction of family planning (23), with the total fertility rate consistently remaining below replacement level (24). Second, the Chinese government has recently issued documents pertaining to fertility policy (25, 26). Finally, as one of the few countries in the world with a population exceeding 1 billion, examining and enhancing China’s fertility policy holds significant implications for improving global fertility trends. In June 2021, the Chinese government issued the “Decision on Optimizing Fertility Policies to Promote the Long-term Balanced Development of Population,” with the objective of encouraging women’s fertility intentions and facilitating the balanced development of the population (27). Following this, various provincial governments in China implemented a range of programs. National-level documents were excluded from the selected policy texts because they are guideline-type materials that influence the evaluation of policy coherence. Additionally, since the assessment focuses on the shortcomings of the policy texts, it is essential to select the most recent policy documents. Based on this, our investigation identified the “three-child policy” and the initiative for “promoting long-term balanced development of population” on websites such as “china.gov.cn,” “Beida Faber,” and “Baidu.” We conducted searches using keywords such as “three-child policy,” “promoting long-term balanced population development,” and “optimizing fertility policy,” ultimately selecting 22 publicly available policy texts from Chinese provincial governments, as detailed in Table 1.

**Table 1:** Selection of policy texts.

Serial no.	Name of policy	Date of issue
1	Circular of the Jilin Provincial People’s Government of the Communist Party of China on the Implementation Plan on Optimizing Fertility Policies to Promote Long-term Balanced Development of the Population	Dec. 2021
2	Circular of the Beijing Municipal Committee of the Communist Party of China and Beijing Municipal People’s Government on the Implementation Plan on Optimizing Fertility Policies to Promote Long-term Balanced Development of Population	Jan. 2022
3	Implementation Plan of the Fujian Provincial People’s Government on Optimizing Fertility Policies to Promote Long-term Balanced Population Development	May. 2022
4	Circular of the General Office of the Guangdong Provincial Party Committee and the General Office of the Guangdong Provincial People’s Government on the Issuance of the Implementation Plan for Optimizing Fertility Policy and Promoting Long-term Balanced Population Development	Dec. 2021
5	Implementation Opinions of the Committee of the Communist Party of China of the Guangxi Zhuang Autonomous Region and the People’s Government of the Guangxi Zhuang Autonomous Region on Optimizing Reproductive Policies to Promote Long-term Balanced Development of the Population	May. 2022
6	Notice issued by the Guizhou Provincial People’s Government of the CPC Guizhou Provincial Committee on the Implementation Plan on Optimizing Reproductive Policies to Promote Long-term Balanced Development of Population	Dec. 2021
7	Circular of the Hainan Provincial Committee of the Communist Party of China and the People’s Government of Hainan Province on the issuance of the Implementation Plan on Optimizing Fertility Policies to Promote Long-term Balanced Development of Population	Jun. 2022
8	Implementation Plan for Optimizing Fertility Policies and Promoting Long-term Balanced Population Development in Henan Province	May. 2022
9	Implementation Plan of the Heilongjiang Provincial Committee of the Communist Party of China and the People’s Government of Heilongjiang Province on Optimizing Fertility Policies to Promote Long-term Balanced Population Development	Apr. 2022
10	Circular of the Hunan Provincial People’s Government of the Hunan Provincial Committee of the Communist Party of China on the Implementation Plan on Optimizing Fertility Policies to Promote Long-term Balanced Population Development	Jun. 2022
11	Circular of the Jiangsu Provincial Committee of the Communist Party of China and the Jiangsu Provincial People’s Government on the Issuance of the Implementation Plan of Jiangsu Province on Optimizing Fertility Policies and Promoting Long-term Balanced Population Development	Feb. 2022
12	Implementation Plan for Optimizing Fertility Policy and Promoting Long-term Balanced Population Development in Jiangxi Province	Jun. 2022
13	Implementation plan of the Autonomous Region’s Party Committee and Government on the optimization of fertility policy for the long-term balanced development of the population	Nov. 2022
14	Circular of the Party Committee of the Ningxia Autonomous Region People’s Government of the Autonomous Region Issuing the Implementation Plan on Optimizing Reproductive Policies to Promote Long-term Balanced Population Development	Jun. 2022
15	Circular of the Shandong Provincial People’s Government of the Shandong Provincial Committee of the Communist Party of China on the Issuance of the Implementation Plan for Optimizing Fertility Policies and Promoting Long-term Balanced Population Development	Sept. 2022
16	Circular of the Shanxi Provincial Committee of the Communist Party of China and the Shanxi Provincial People’s Government on the Implementation Plan on Optimizing Fertility Policies to Promote Long-term Balanced Population Development	Jan. 2023
17	Implementation Plan for Optimizing Reproductive Policies and Promoting Long-term Balanced Population Development in Shaanxi Province	Dec. 2021
18	Circular of the Committee of the Xinjiang Production and Construction Corps of the Communist Party of China on the Implementation Plan on Optimizing Fertility Policies to Promote the Long-term Balanced Development of the Population, issued by the Xinjiang Production and Construction Corps.	May.2022
19	Yunnan Provincial Committee of the Communist Party of China Yunnan Provincial People’s Government Issues Implementation Plan on Optimizing Reproductive Policies to Promote Long-term Balanced Population Development	Sept. 2022
20	Implementing Opinions of the Zhejiang Provincial People’s Government on Optimizing Fertility Policies to Promote Long-term Balanced Population Development.	Jun. 2022
21	The Chongqing Municipal Committee of the Communist Party of China and the Chongqing Municipal People’s Government issued the Implementation Plan on Optimizing Fertility Policies to Promote Long-term Balanced Population Development	Jul. 2022
22	Circular of the Communist Party of China Gansu Provincial Committee and the People’s Government of Gansu Province on the issuance of the Implementation Plan on Optimizing Fertility Policies to Promote the Long-term Balanced Development of the Population	Dec. 2022

### 2.2 Model building

#### 2.2.1 Keyword semantic network construction

In accordance with the aforementioned policy text, the initial screening of the research object involved the removal of irrelevant information, such as “document number,” “title,” and “time of issuance.” Subsequently, the policy texts of the 22 selected policies were compiled into TXT files, and text mining was conducted using the ROSTCM6 software (28, 29). Among them, the high-frequency keywords are based on the following three cleaning principles. Firstly, the top 100 high-frequency keywords are selected, and highly relevant terms such as “birth” and “policy” are filtered out. Secondly, we filtered out meaningless verbs such as “enhance,” “strengthen,” “carry out,” etc. Finally, we filtered out auxiliary words such as “of” and “on.” A list of the top 20 high-frequency keywords is formed, as shown in Table 2.

**Table 2:** Table of high-frequency keywords.

No.	Vocabulary	Word frequency	No.	Vocabulary	Word frequency
1	Service	1,099	11	Reproduction	117
2	Household	516	12	Health	116
3	Organization	338	13	Housing	112
4	System	282	14	Supplement	103
5	Infant	229	15	Technical	103
6	Inclusive childcare	187	16	Maternal and child health	101
7	Measure	184	17	Nursery school	98
8	Education	181	18	Ancillary service	95
9	Care	155	19	Pension	88
10	Cultivate	130	20	Insurances	88

**Figure 2:**
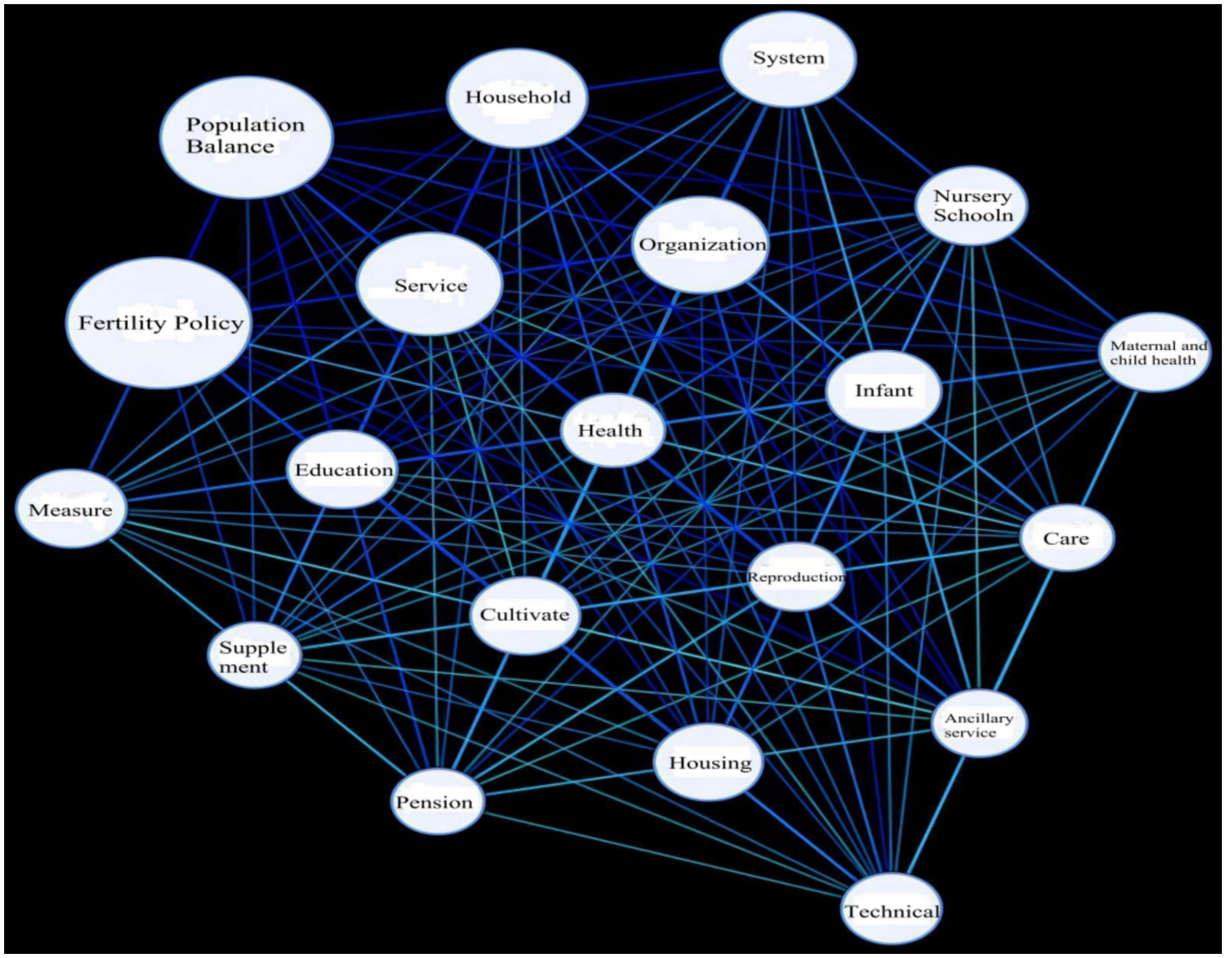
Semantic network diagram of high-frequency words.

Subsequently, we constructed a semantic network diagram of core keywords based on the high-frequency keyword list (30), as illustrated in Figure 2. In this semantic network, the core keywords situated in the upper left of the diagram are “fertility policy” and “population balance,” while the remaining keywords are organized around these core terms. The size of each keyword’s circle indicates its closeness to other keywords, and the length of the connecting lines reflects the degree of association between subwords; shorter lines signify a closer connection. Notably, the keywords “family,” “service,” “education,” and “measures” represent areas where connections should be strengthened within the policy framework. Therefore, these aspects should be prioritized in the establishment of the multi-input scale.

#### 2.2.2 Multi-input–output scale construction

Based on an analysis of keywords derived from high-frequency words and the mapping of social network relationships, and in accordance with Estrada’s policy evaluation criteria (15, 31), we developed a PMC fertility policy evaluation model that utilizes text mining techniques. This model comprises nine primary variables and 41 secondary variables, as detailed in Table 3.

**Table 3:** Indicators of policy evaluation variables.

Level 1 variable	Secondary variables
Target validity period $X_{1}$	Short-term $X_{11}$ ; Mid-term $X_{12}$ ; Long-term $X_{13}$
Nature of the policy $X_{2}$	Anticipate $X_{21}$ ; Supervisory $X_{22}$ ; Suggestion $X_{23}$ ; Descriptive $X_{24}$ ; Introduction $X_{25}$
Policy area $X_{3}$	Politically $X_{31}$ ; Economics $X_{32}$ ; Social security $X_{33}$ ; Regime $X_{34}$
Policy target $X_{4}$	Establishment of a policy system $X_{41}$ ; Clarify the main body of responsibility $X_{42}$ ; Complete management system $X_{43}$ ; Reinforcement of follow-up guarantees $X_{44}$ ; Building service systems $X_{45}$
Policy content $X_{5}$	Implementing family maternity leave $X_{51}$ ; Improving service levels $X_{52}$ ; Developing inclusive childcare $X_{53}$ ; Reducing the cost of childbirth $X_{54}$ ; Enhancing policy alignment $X_{55}$ ; Strengthening implementation safeguards $X_{56}$
Policy evaluation $X_{6}$	Well-founded $X_{61}$ ; Clarity of purpose $X_{62}$ ; Well planned $X_{63}$ ; Options feasible $X_{64}$
Incentive protection $X_{7}$	Financial input $X_{71}$ ; Subsidized incentives $X_{72}$ ; Pilot demonstrations $X_{73}$ ; Infrastructures $X_{74}$ ; Complementary measure $X_{75}$ ; Employment protection $X_{76}$ ; Organizational implementation $X_{77}$ ; Legal protection $X_{78}$
Policy receptor $X_{8}$	Governments $X_{81}$ ; Corporations $X_{82}$ ; The masses $X_{83}$
Policy tools $X_{9}$	Demand-based $X_{91}$ ; Supply-based $X_{92}$ ; Environment-based $X_{93}$
Policy disclosure $X_{10}$	No secondary variables

Multi-input–output scales are constructed based on the above indicators of policy evaluation variables, as shown in Table 4.

**Table 4:** Multiple input–output scale.

Primary variables	X1	X2	X3	X4	X5	X6	X7	X8	X9	X10
Secondary variables	X1:1	X2:1	X3:1	X4:1	X5:1	X6:1	X7:1	X8:1	X9:1	-
	X1:2	X2:2	X3:2	X4:2	X5:2	X6:2	X7:2	X8:2	X9:2	
	X1:3	X2:3	X3:3	X4:3	X5:3	X6:3	X7:3	X8:3	X9:3	
		X2:4	X3:4	X4:4	X5:4	X6:4	X7:4			
		X2:5		X4:5	X5:5		X7:5			
					X5:6		X7:6			
							X7:7			
							X7:8			

## 3 Results

### 3.1 PMC index calculation

The PMC index is calculated by assessing the evaluation indicators established above using a multi-input scale. Initially, values of 0 and 1 are assigned to each index. Subsequently, the values of secondary variables are computed, and based on their summation along with the primary variables, a 3×3 PMC matrix is constructed (32, 33). The formula is shown in Equations 1–3:


(1) $$X_{ij}\sim N\left(0,1\right)$$

(2) $$X_{i}\left(\overset{n}{\underset{j=1}{\sum }}\frac{X_{i-j}}{n}\right)$$

(3) $$PMC=\left[\begin{array}{c} X_{1}\left(\overset{3}{\underset{t=1}{\sum }}\frac{X_{1t}}{3}\right)+X_{2}\left(\overset{5}{\underset{j=1}{\sum }}\frac{X_{2j}}{5}\right)+X_{3}\left(\overset{4}{\underset{k=1}{\sum }}\frac{X_{3j}}{4}\right)+\\ \;X_{4}\left(\overset{5}{\underset{t=1}{\sum }}\frac{X_{4t}}{5}\right)+X_{5}\left(\overset{6}{\underset{m=1}{\sum }}\frac{X_{5m}}{6}\right)+X_{6}\left(\overset{4}{\underset{n=1}{\sum }}\frac{X_{6n}}{4}\right)+\\ X_{7}\left(\overset{8}{\underset{o=1}{\sum }}\frac{X_{7o}}{8}\right)+X_{8}\left(\overset{3}{\underset{p=1}{\sum }}\frac{X_{8p}}{3}\right)+X_{9}\left(\overset{3}{\underset{q=1}{\sum }}\frac{X_{9q}}{3}\right)+X_{10}\\ \; \end{array}\right]$$

And based on the PMC rating criteria indicated by Estrada, the policies were categorized into perfect, excellent, acceptable, and bad policies (15, 30) as shown in Table 5.

**Table 5:** Policy evaluation ratings.

PMC index	9–10	7–8.999	5–6.999	0–4.999
Rating	Perfect policy (PP)	Good policy (GP)	Acceptable policy (AP)	Low policy (LP)

To calculate the PMC index for 22 policies, we first discriminate the secondary variable $X_{ij}$ based on the policy text. If the policy text includes the conditions of the secondary variable, it is assigned a value of 1; otherwise, it is assigned a value of 0. Subsequently, we calculate the primary variable, leading to the determination of the PMC index for each policy. The specific results of these calculations are presented in Tables 6, 7.

**Table 6:** Calculation of PMC index.

Level 1 variable	P1	P2	P3	P4	P5	P6	P7	P8	P9	P10	P11
X1	0.33	0.33	0.33	0.33	0	0.33	0.33	0.33	0.33	0.33	0.33
X2	0.8	0.6	0.6	0.6	0.8	1	0.8	0.8	0.6	0.8	0.6
X3	1	1	1	1	1	1	1	1	1	1	1
X4	0.8	0.8	0.8	0.8	0.8	1	0.8	0.8	0.8	0.8	0.8
X5	0.84	0.68	0.84	1	0.68	1	0.84	0.84	0.84	1	0.84
X6	1	1	1	0.75	0.75	1	1	1	1	1	1
X7	0.75	0.75	0.625	0.625	0.625	0.75	0.625	0.75	0.75	0.875	0.75
X8	0.67	0.67	1	0.67	0.67	0.67	0.67	0.67	0.67	0.67	1
X9	0.33	0.33	0.33	0.33	0.33	0.33	0.33	0.33	0.33	0.33	0.33
X10	1	1	1	1	1	1	1	1	1	1	1
PMC index	7.52	7.16	7.53	7.11	6.66	8.08	7.4	7.52	7.32	7.81	7.65
Rating	GP	GP	GP	GP	AP	GP	GP	GP	GP	GP	GP
PMC ranking	12	16	11	17	22	2	13	8	14	3	7

**Table 7:** Calculation of PMC index.

Level 1 variable	P12	P13	P14	P15	P16	P17	P18	P19	P20	P21	P22
X1	0.33	0.33	0.33	0.33	0.33	0	0.33	0.33	0.33	0.33	0.33
X2	0.8	0.8	0.6	0.6	0.8	0.8	0.6	1	0.8	0.8	0.6
X3	1	1	1	1	1	1	1	1	1	1	1
X4	0.8	0.8	1	0.8	0.8	0.8	1	1	0.8	0.8	0.8
X5	0.84	0.84	0.84	0.68	1	0.68	1	1	0.68	1	0.68
X6	1	1	0.75	0.75	1	0.75	1	1	0.75	1	0.75
X7	0.75	0.75	0.75	0.75	0.75	0.75	0.875	0.875	0.5	0.875	0.625
X8	0.67	0.67	0.67	0.67	0.67	0.67	0.67	0.67	0.67	0.67	0.67
X9	0.33	0.33	0.33	0.33	0.33	0.33	0.33	0.33	0.33	0.33	0.33
X10	1	1	1	1	1	1	1	1	1	1	1
PMC index	7.52	7.52	7.27	6.91	7.68	6.78	7.81	8.21	6.86	7.81	6.79
Rating	GP	GP	GP	AP	GP	AP	GP	GP	AP	GP	AP
PMC ranking	8	8	15	18	6	21	3	1	19	3	20

### 3.2 PMC surface drawing

**Figure 3:**
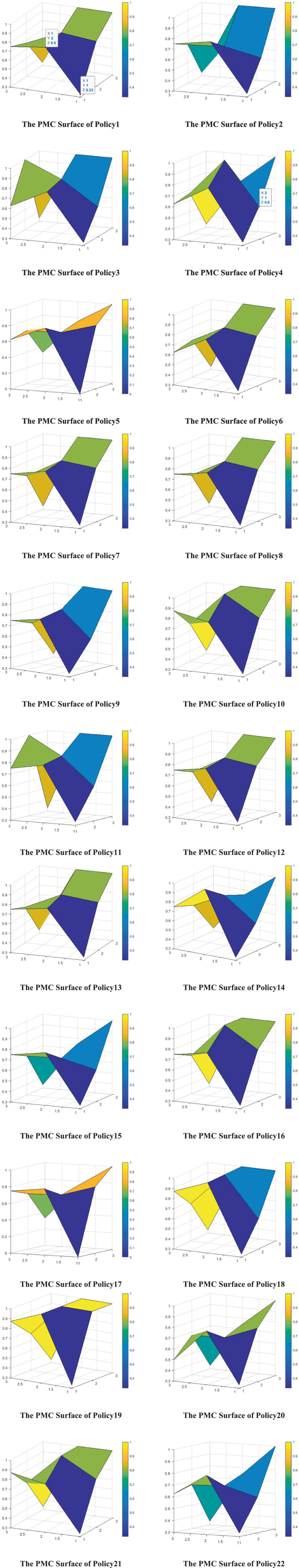
22 Policy PMC Surfaces.

The PMC surface map serves as a three-dimensional visual assessment tool that facilitates a more intuitive visualization of the strengths and weaknesses of each policy, guided by the PMC concavity index. The PMC surface diagram is represented as a 3×3 matrix. Instead of a 10×10 matrix, we introduce a first-level variable; all documents considered are public policies, rendering the inclusion of a larger matrix impractical. Consequently, in constructing the PMC surface diagram, we eliminate this larger matrix, ultimately resulting in a three-dimensional surface diagram. The transition from yellow to blue indicates varying scores for each item, with darker colors representing lower scores. The specific scores correspond to the surface diagram side legend, as illustrated in Figure 3.

An analysis of the PMC surfaces of the 22 policies reveals both their weaknesses and strengths. Overall, the performance of these policies is subpar in the X1, X8, and X9 indicators, indicating a lack of foresight regarding the target period. The policies appear to be primarily designed based on recent conditions, without a thorough examination and assessment of medium- and long-term demographic changes. Furthermore, the indicators related to policy recipients and instruments suggest that the fertility policy documentation inadequately addresses the full spectrum of methodologies and requires further enhancement.

In this discussion, we have selected representative policies for in-depth analysis. The first policy under consideration is Policy19, which is ranked No. 1. According to the calculation of the PMC index, Policy19 boasts a value of 8.21, categorizing it as an excellent policy text. The first-level indicators reveal that Policy19 excels in indicators X2, X3, X4, X5, and X6, suggesting it offers comprehensive coverage and rich content regarding policy areas, objectives, content, and evaluation. Conversely, the indicators influencing its index are X1, X8, and X9. The three key elements are target duration, policy receptors, and policy instruments. Regarding performance, the duration indicator is absent because Policy 19 does not specify an expectation for achieving the target. Additionally, the indicator for policy receptors is lacking, as Policy 19 fails to consider the risks faced by enterprises. Relevant studies indicate that enterprises may incur higher fertility costs due to the influence of the three-child policy (34). This underscores the importance of including enterprises in the policy framework. Lastly, concerning the indicators for policy instruments, Policy 19 does not adequately address public demand or the overall fertility environment, highlighting a need for further refinement in the policy text. In light of these observations, we propose a policy enhancement pathway of X1 → X9 → X8 for Policy 19.

For the second analysis, we selected the lowest-ranked Policy5. According to the PMC index calculation, Policy5 has a PMC index value of 6.66, indicating that it is an acceptable policy text. However, at the first level, Policy5 performs poorly on indicators X1, X5, X6, X7, X8, and X9, necessitating further enhancement. Specifically, regarding X1, Policy5 lacks a temporal plan for implementation. Additionally, in relation to X5, X6, and X7, Policy5 contains relatively few policy texts and does not comprehensively address various aspects of fertility policy, particularly in the areas of “family” and “services,” as identified through our text mining analysis. The concept of “family” plays a significant role in influencing maternity hospitals (35). A study published in Nature indicates that the UK’s maternity leave policy should be designed to benefit the entire family (36), highlighting that Policy 5 lacks comprehensive coverage and requires supplementation. Additionally, the X8 and X9 indicators are relatively weak among the 22 policy texts and warrant further strengthening. Consequently, we propose a policy enhancement pathway of X1 → X9 → X7 → X8 → X5 → X6 for Policy 5.

Thirdly, we have selected Policy3, which is ranked 11 th. According to the PMC index calculation, the PMC index value of Policy3 is 7.53, indicating that it is an excellent policy text. Analyzing the specific scores reveals that Policy3 exemplifies a typical policy document, primarily relying on the macro-indicators mandated by the state, while neglecting to explore deeper aspects. This limitation is particularly evident in indicators X2 and X7, where Policy3 offers policy guidance in key areas of national concern but lacks relevant guidelines and expectations, and the safeguards for the policy are insufficient and require further strengthening. In light of this, we propose a policy enhancement pathway of X1 → X9 → X2 → X8 → X7 → X5 for Policy3.

## 4 Conclusion and policy implications

### 4.1 Conclusion

This study investigated a total of 22 Chinese government fertility policy texts and conducted policy evaluation based on a quantitative fertility policy assessment model for text mining, and drew the following conclusions:

(1) China’s fertility policy texts are relatively comprehensive in design, with an overall PMC index ranging from 6.66 to 8.21, which contains 17 excellent policy texts, 5 acceptable policy texts, and a relatively high overall policy text consistency.
(2) There is still much room for improvement in the current fertility policy texts. Based on the above PMC surfaces of the 22 Chinese fertility policies, the overall degree of policy concavity is large, especially in the medium- and long-term expectations of the policies as well as the coverage of the policies. Therefore, it is necessary to focus on the expectations of the policies, strengthen the duration of the policies, and comprehensively consider all factors affecting the fertility policies, so as to promote the high-quality development of the fertility policies.
(3) The degree of refinement of the policy still needs to be increased. In the text of the policy, only intuitive factors such as costs and childcare are taken into account, and the indirect factors that increase fertility intentions are not comprehensively considered, whereas the essence of promoting balanced development of the population is to promote women’s fertility intentions. Therefore, relevant policies should take more account of factors such as “family,” “subsidies,” and “occupation” (37–39). For example, the implementation of paid parental leave (40), to increase the status of women’s occupation (41), etc., only better protection of women’s rights, make families happier, and only in a real sense to achieve the enhancement of fertility intentions.

### 4.2 Policy impact

China’s current fertility policy is still insufficient in terms of segmentation. In order to promote the balanced development of China’s population, prevent labor shortages, food security and other social-economic problems caused by population shortages (42), and solve the problems of insufficient coverage and lack of balance in the text of the current fertility policy, the following countermeasures are proposed:

(1) Strengthening cooperation among multiple subjects

Based on the current policy texts issued by governments at all levels, we find that the government is still planning from a macro perspective, but micro situations such as promoting women’s fertility intentions are difficult to cover comprehensively from a macro perspective alone. Therefore, policy issuers should co-operate with social and grass-roots organizations to jointly plan appropriate promotion measures, and if necessary, set up a multi-dimensional linkage mechanism to strengthen the co-operation between the government and social capital organizations to jointly evaluate the policy texts.

(2) Promoting multi-objective development

Based on the dynamic assessment of the current policy text, we find that the balance of the policy text is weak, and there are flaws in some key elements of public concern. In the process of the government’s promotion of balanced population development, it should synergize multi-objective development, with incentives as the main means to seek benefits and treatment for families of childbearing age. From the economic aspect, it should protect the future costs of education, life, childbirth and childcare for families of childbearing age; from the policy aspect, it should increase maternity and paternity leave for families of childbearing age; and from the aspect of future careers, it should raise the rank or treatment of both parents who have children, so as to achieve the purpose of promoting the development of fertility intentions.

(3) Changing the concept of reproductive culture

The effective promotion of fertility is never something that can be fully resolved by a single policy, but should be promoted from a fundamental perspective, which is what the current text of the fertility policy needs to be improved. The fundamental promotion of fertility intentions is actually a change in cultural concepts, the current social development of the fast era, the pursuit of profit among families, so as to achieve the maximization of the economy, and the birth of children is contrary to this, therefore, the dissemination of traditional culture, the concept of child-rearing to change is the key to the realization of the high quality of the development of fertility intentions.

In summary, this paper presents an empirical study of China’s 22 fertility policies, which holds significant importance for guiding the revision of fertility policy texts and plays a crucial role in promoting the long-term equilibrium of the population. However, several issues within this paper warrant further investigation:

1. Fertility policy is a newly introduced policy framework in China, yet it is widely recognized that fertility policies exert long-term effects. Consequently, ongoing research is essential to monitor these policies, and future studies will be conducted to better inform the development of effective measures that influence fertility trends.
2. Fertility policy is largely shaped by women’s preferences, and while the policy text can facilitate these preferences, it cannot dictate them. Therefore, future research should consider integrating both subjective and objective approaches to enhance relevance to the social context.

## Data Availability

The raw data supporting the conclusions of this article will be made available by the authors, without undue reservation.
